# Granular cell odontogenic tumor: report of a rare case and a review of literature

**DOI:** 10.1186/s12903-022-02097-2

**Published:** 2022-03-05

**Authors:** Milad Etemadi Sh, Sayed Mohammad Razavi, Olia Ghazavi, Mohammad Hossein Nikbakht

**Affiliations:** 1 Department of Oral and Maxillofacial Surgery, Dental Implants Research Center, Dental Research Institute, School of Dentistry, Isfahan University of Medical Sciences, Isfahan, Iran; 2 Department of Oral and Maxillofacial Pathology, Dental Implants Research Center, School of Dentistry, Isfahan University of Medical Sciences, Isfahan, Iran; 3 Student Research Committee, School of Dentistry, Isfahan University of Medical Sciences, Isfahan, Iran

**Keywords:** Granular cell tumor, Odontogenic tumors, Oral cavity, Mandible, Case report

## Abstract

**Background:**

Granular cell odontogenic tumor (GCOT) is a rare neoplasm with about 45 cases reported in the literature. It usually occurs in the posterior mandible of middle-aged women.

**Case presentation:**

We report a case of asymptomatic GCOT in the posterior mandible of a 28 years old female and provide a literature review of GCOT cases. Some unusual features such as root resorption, displacement of inferior tooth canal, and multilocular appearance were considerable in this case.

**Conclusions:**

Complete surgical excision of the lesion was beneficial for the patient.

## Background

Granular cell odontogenic tumor (GCOT) is a rare benign neoplasm of jaws [1] which was first described with the term “spongiocytic adamantinoma” by Werthemann in 1950 [2]. This lesion was also called “Granular cell ameloblastic fibroma” and “granular cell odontogenic fibroma”. It usually occurs in the posterior region of the mandible with definite female predilection in the fifth decade of life [1]. Approximately 45 cases were reported in the literature [1, 3–9]. GCOT usually presents as an asymptomatic painless swelling in tooth-bearing areas of the jaws [10], so they are often found accidentally in routine radiographs [11]. Radiographs usually show Unilocular radiolucent lesions with sclerotic borders, but sometimes they are multilocular or mixed radiolucent-radiopaque. Small dystrophic calcifications can be seen in some cases [1, 10]. Histopathologically, GCOT consists of sheets and lobules of large eosinophilic granular cells immersed in a fibroblastic stroma. Small islands or narrow cords of odontogenic epithelium are scattered among the granular cells [1, 12]. The differential diagnosis of GCOT from granular cell ameloblastoma, granular cell tumor, and congenital epulis of the newborn is important [13].

The prognosis of GCOT is good and it responds well to curettage [14]. Only one case of malignancy [14] and 2 cases of recurrence were reported in the literature [11, 15]. No case of metastasis has been reported. However, it is necessary to follow-up the patient evaluate the long-term outcome [1]. This study aims to describe a rare case of difficult diagnosis mandibular GCOT.

## Case presentation

We have read the Helsinki Declaration and have followed the guidelines in this investigation.

**Fig. 1:**
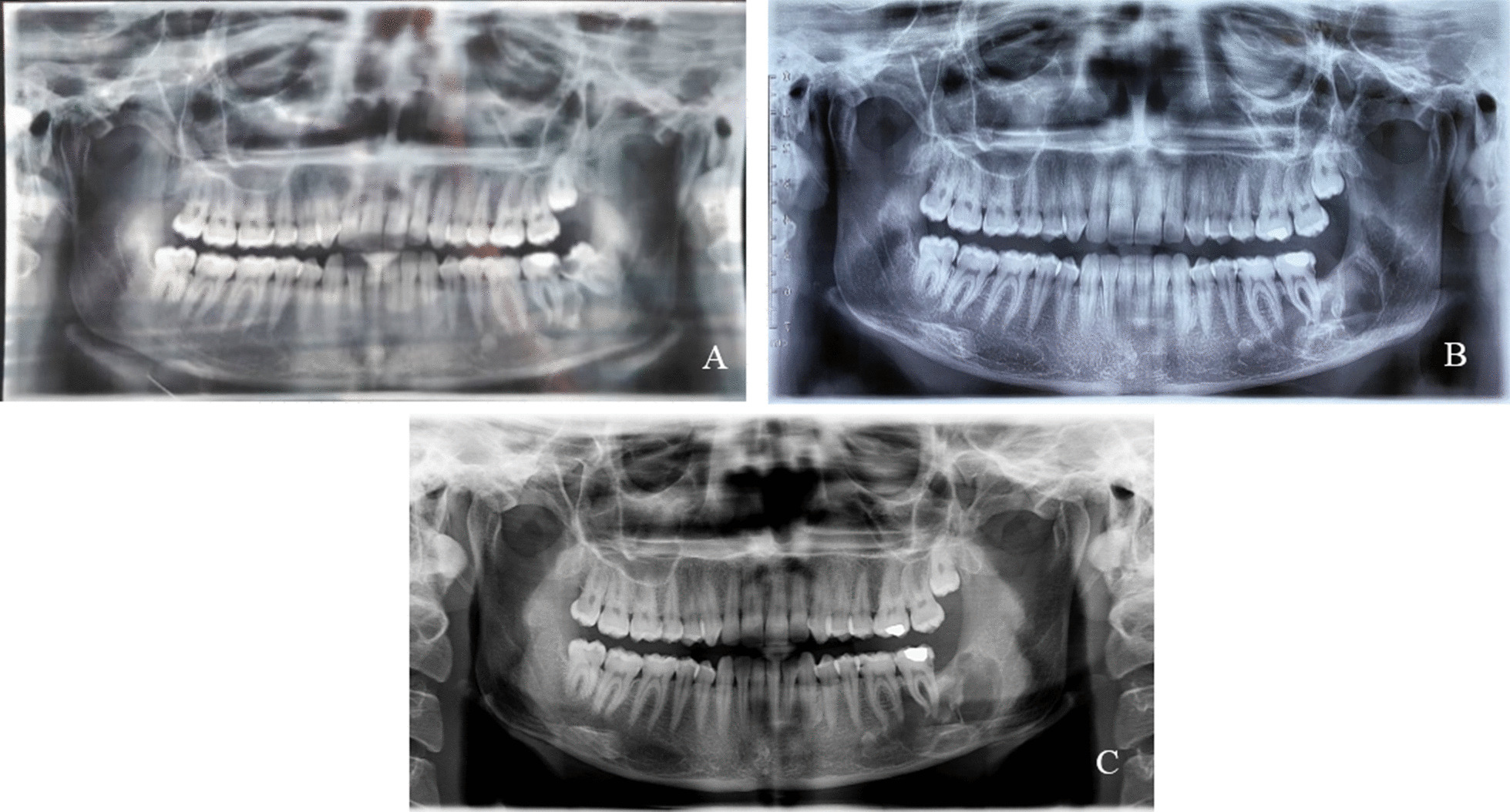
**A** Orthopantomography demonstrating multilocular radiolucency, **B** Orthopantomogram taken 3 months post-operatively. The lesion site is undergoing a healing process, **C** Orthopantomogram taken 6 months post-operatively. Notice the healing process and shrinkage of the lesion

**Fig. 2:**
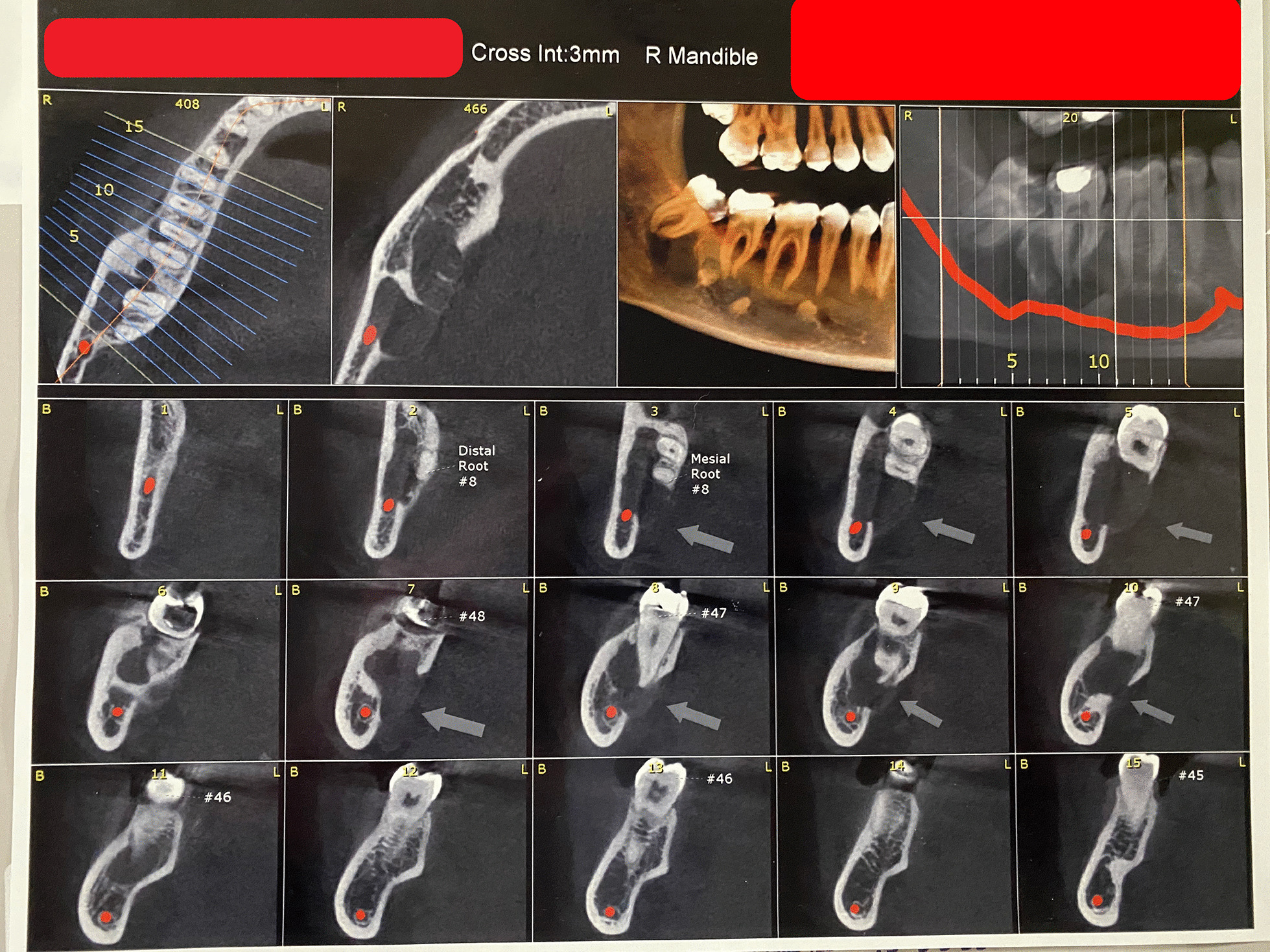
CBCT image

**Fig. 3:**
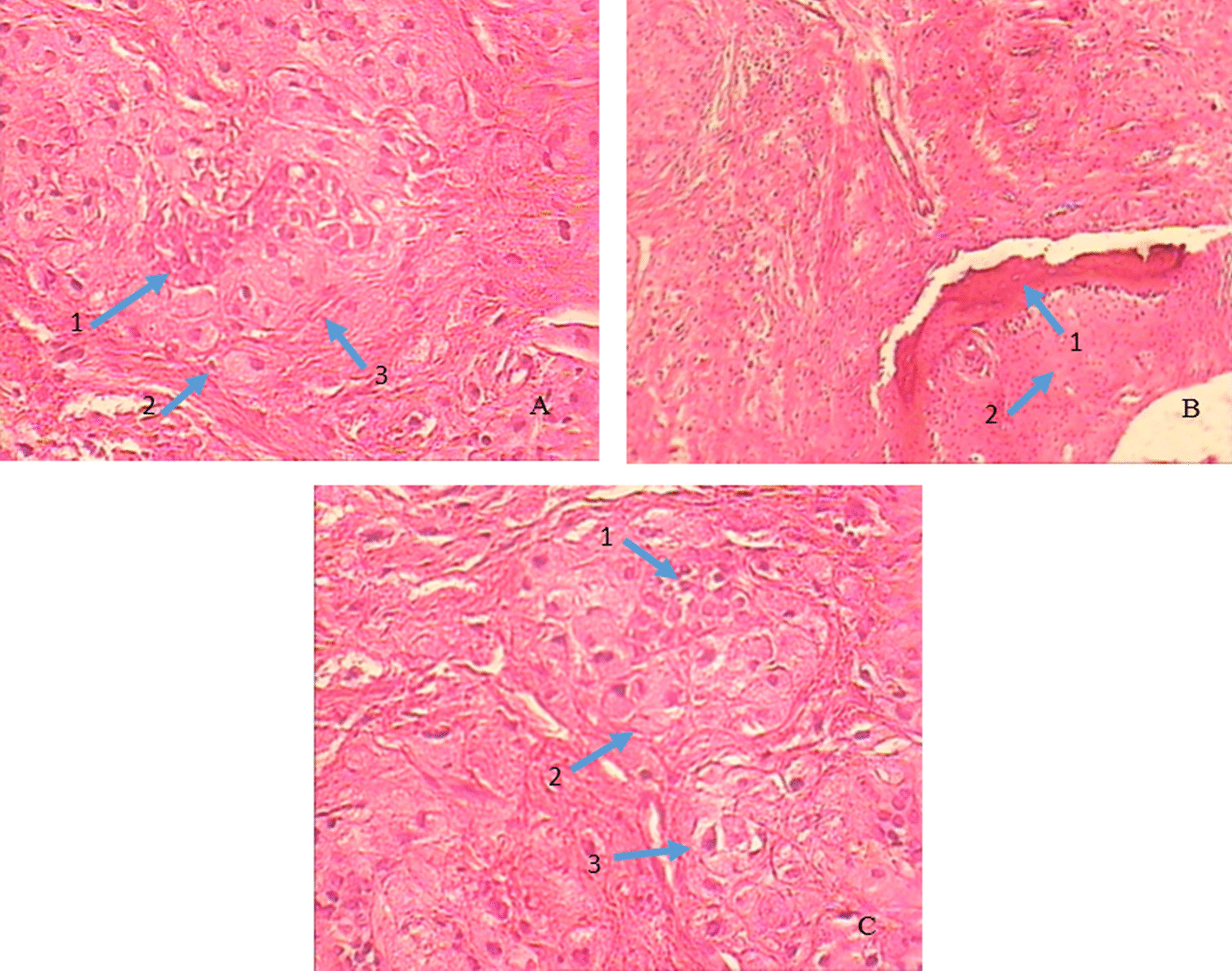
Photomicrograph shows, **A** odontogenic epithelium colony adjacent to the granular cells, 1: Odontogenic epithelial islands 2, 3: Sheets of mesenchymal granular cells (H&E × 40). **B** 1: Dystrophic calcification near the granular cells 2: Sheets of mesenchymal granular cells (H&E × 100) **C** 1: Odontogenic epithelial islands 2, 3: Sheets of mesenchymal granular cells (H&E × 400). Olympus CX43 microscope and Canon DSLR EOS 1300D were used for the images and no downstream processing or averaging were performed

**Fig. 4:**
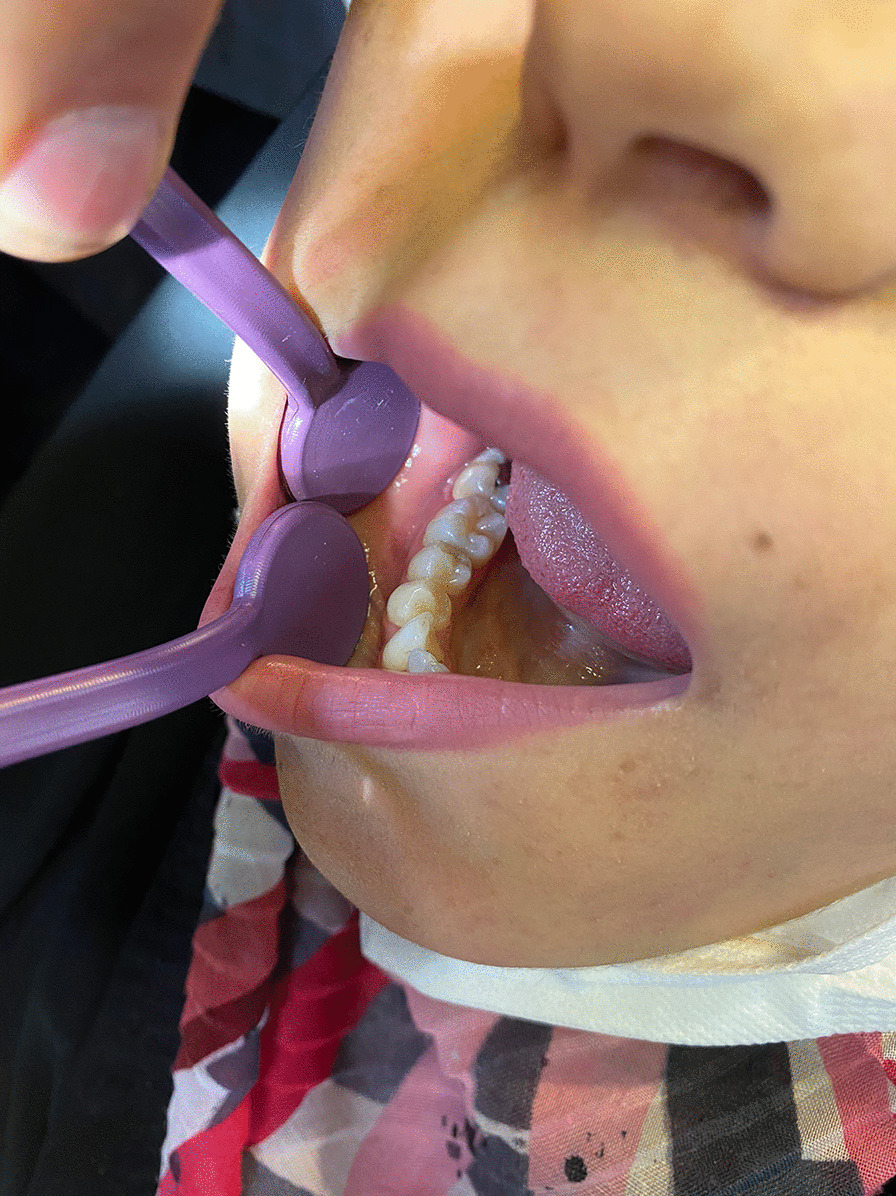
Clinical image of the case 6-months after surgery

A 28 years old woman with a painless carious wisdom tooth in her right mandible attended a dental clinic. A general dentist visited her and Orthopantomography (OPG) radiographs (Fig. 1) were taken. Accidently a multilocular radiolucent lesion was observed in her right mandible, so she was referred to an oral and maxillofacial surgeon in Isfahan school of dentistry. Intraoral examination showed no specific intra-oral sign except a mild sensitivity in the area. There was no pain or swelling. The general head and neck examination, the lymph nodes, and the oral mucosa were normal. She had no history of medical problems. We performed cone-beam computed tomography (CBCT). On CBCT examination (Fig. 2), a multilocular lesion with well-defined cortical borders and the scalloped view was observed adjacent to the first, second, and third molar. The size of the lesion was 12 × 15 × 30 mm and it had been manipulated to the inter-radicular regions of molar teeth. The lesion led to the destruction of lamina dura of adjacent teeth, resorption of the lingual plate of alveolar bone, and displacement of inferior tooth canal towards down. Multiple radiopaque regions of compact bone were observed in the buccal and lingual surface of the lesion, attached to the bone cortex. The third and second molars had advanced decay, respectively. Dental plaque and gingivitis were observed. According to the clinical and radiographic findings, radicular cyst of 3rd molar, Odontogenic keratocyst (OKC), Mural ameloblastoma, Calcifying odontogenic cyst (COC), and dentigerous cyst were considered as the differential diagnosis. The wisdom tooth was extracted and the lesion completely excised under sedation. The excisional specimen arrived for pathological examination as multiple soft tissue fragments measuring up to 10 × 15 × 25 mm in brown cream color. Histopathologic examination (Fig. 3). They revealed proliferation of eosinophilic granular cell sheets, in which islands or narrow cords of odontogenic epithelium and multinuclear giant cells were scattered. Dystrophic calcification centers were also seen in some areas. There was no evidence of malignancy in the lesion; therefore no additional treatment was performed. Due to the findings, the diagnosis of (GCOT) was made. The patient was followed and after 3 months the lesion site was undergoing a healing process and after 6-months shrinkage of the lesion was observed (Figs. 1, 4). No recurrence had happened.

## Discussion and conclusions

The review of the literature shows, to date, 46 cases of GCOT have been reported including the current case. The information presented in Table 1 is extracted from these cases [1, 3–9]. The age range of GCOT is 16–77 years with an average of 43.91 years (Table 1). GCOT mostly occurs in the 5th to 7th decades of life [1], however, the age of the presented case was 28 years old which is 16 years lesser than the average. Similar to most cases which show the female to the male distribution of 3.5:1 (Table 1), the presented case is female.

**Table 1:** Clinicopathologic details of reported cases of central granular cell odontogenic tumor

Total case number	46
Age	Range: 16–77 years, average: 43.91 years
Sex	Male: 10, female: 35
Location	Mandible: 34, maxilla: 10
	Posterior: 31, anterior: 4, anterior–posterior: 5
	Left: 19, right: 14

In the presented case, the lesion was located in the posterior region of the mandible which is the most common site of GCOT with a mandible to maxilla ratio of 3.4:1 (Table 1). GCOT tends to occur a little more on the left side (57.6%) rather than on the right side (42.4%) (Table 1). However, this tendency applies to the tumors of the mandible, but not the maxilla [15]. In the current case, the tumor was on the right side which was contrary to most previous studies.

GCOT usually occurs as an asymptomatic lesion with slowly growing swelling [1]. The current case was also asymptomatic and didn’t have any pain or swelling as same as the case that Lee et al. [7] reported. In some studies, cases had just pain and no swelling [5, 8] or vice versa [3, 4, 6, 9].

Calcifications have been found in 50% of the reported cases of GCOT [1, 3–9]. Dystrophic calcification centers were seen in the current case. Cortical expansion, displacement of teeth, cortical perforation, and displacement of the mandibular canal were also seen in some cases [1]. Root resorption was seen in the current case which was only reported in 5 other cases [1, 3, 5, 6]. Displacement of the inferior tooth canal was seen in the presented case which Couch et al. and Vincent et al. have also reported [16, 17]. Most of the studies reported unilocular radiographic appearance of the lesion [1]. However, some cases showed multilocular appearance which is also seen in the current case [1, 4].

The average size of the lesion is 2.8 cm which ranges from 0.5 to 8 cm [1]. The lesion size of the present case was 3 cm. On gross examination, the lesion usually consists of whitish-colored tissues [1]. However, in the reported case the specimen was received in brown cream color tissue fragments.

Due to the clinical and radiographic findings, the differential diagnosis consists of radicular cyst of 3rd molar, OKC, Mural ameloblastoma, COC, and dentigerous cyst. Since these findings are not sufficient for an accurate diagnosis, histopathological examination is necessary. It is very difficult to distinguish OKC from GCOT based on clinical view and radiography, but it can be said that OKC is more common in the posterior mandibular region, while GCOT is more common in the premolars. In clinical view differential diagnosis of Mural unicystic ameloblastoma from GCOT isn’t possible but in GCOT radiographs may show small calcifications. Based on the clinical picture, it is not possible to distinguish COC from GCOT, but in the radiographic view of COC, radiopaque areas may be seen, while in many cases it occurs with an impacted tooth that is mostly canine, but in GCOT radiolucency of one or more foci can bee seen and the affected teeth are fully grown. Diagnosis of dentigerous cyst from GCOT is not possible based on clinical view, but the radiological view of dentigerous cyst occurs as a pericoronal radiolucency of an impacted or semi-impacted tooth, but GCOT occurs as unilocular and multilocular radiolucency inside or around the teeth roots [18].

The histopathologic differential diagnosis of GCOT includes granular cell ameloblastoma (GCA), granular cell tumor (GCT), and congenital epulis of the newborn [13]. Immunoreactivity of granular cells (GCs) in GCA for S-100 protein remains inconclusive. However, their immunopositivity for cytokeratin can distinguish them from GCOT [1]. Histopathologically, in the GCOT, a background of granular cells is seen, in which strips, islets, or narrow cords of odontogenic epithelium are located. But in GCA, a background of islets or ameloblastoma follicles is seen and in their center, the stellar reticulum with a round nucleus and granular cytoplasm can be seen [19]. Granular cells of GCT have similarities to those of GCOT, but they show strong immunoreactivity for the S-100 protein in contrast to GCOT. Also, odontogenic islands, cementum-like material, or dystrophic calcifications can’t be seen in GCT [1]. Since the tumor cells in congenital epulis of the newborn are immunonegative for S-100 protein and cytokeratin similar to the GCOT, these markers can’t help distinguish it from GCOT. However, we can distinguish between these two tumors noticing that congenital epulis of the newborn usually occurs in the alveolar ridges of the newborns. Moreover, neuron-specific enolase (NSE) is positive in congenital epulis unlike GCOT [1].

All previous cases were treated through excision and/or curettage [15]. In the present case, the tooth was extracted and excisional surgery was performed. Although the follow-up data shows GCOT has a benign behavior [1], one case of malignancy [14] and 2 cases of recurrence were reported in the literature [11, 15].

In conclusion, GCOT is a rare tumor that can be completely asymptomatic. Hence, it is important to consider GCOT as a possible diagnosis and not to miss it.

## Data Availability

The datasets used and/or analyzed during the current study are available from the corresponding author on reasonable request.

## Abbreviations

GCOT: Granular cell odontogenic tumor
OPG: Orthopantomography
CBCT: Cone-beam computed tomography
OKC: Odontogenic keratocyst
COC: Calcifying odontogenic cyst
GCA: Granular cell ameloblastoma
GCT: Granular cell tumor
GCs: Granular cells
NSE: Neuron-specific enolase
